# Reconstructing impaired language using generative AI for people with aphasia

**DOI:** 10.1038/s41598-025-24725-x

**Published:** 2025-11-19

**Authors:** Achini Adikari, Damminda Alahakoon, Nuwan Pallewela, John E. Pierce, Nelson J. Hernandez, Miranda L. Rose

**Affiliations:** 1 Centre for Data Analytics and Cognition, La Trobe Business School, La Trobe University, Melbourne, Australia; 2 Centre of Research Excellence in Aphasia Recovery and Rehabilitation, La Trobe University, Melbourne, Australia; 3 School of Allied Health, Human Services and Sport, La Trobe University, Melbourne, Australia

**Keywords:** Aphasia, Langchain, GPT, Assistive communication, LLMs for aphasia, Artificial intelligence, Quality of life, Machine learning, Rehabilitation

## Abstract

In an era of Generative Artificial Intelligence (AI), it may be possible to capitalise on AI’s generative capabilities to assist people in compensating for their impaired language. Large Language Models (LLMs) have emerged as a recent breakthrough, revealing the potential to generate fluent, contextually relevant, and coherent texts. The current study leverages this inherent capability of LLMs in text generation and completion to compensate for impaired language in adults with acquired communication disabilities. To date, research studies on LLM for aphasia (a language-based communication disability after brain injury) have focused on specific and well-defined tasks and contexts (e.g., story retelling), and therefore may be less accurate and reliable in real-life conversation scenarios. This research proposes a language-assistive solution embedded in dialogue systems for individuals with aphasia to detect and correct errors in their aphasic speech during natural conversations. We have customised using in-context few-shot prompting (no weight updates) LLM to correct neologisms, paraphasic errors, and word-finding gaps that occur in aphasic speech. This could assist in identifying such errors in conversation and suggest completions in fragmented sentences. We utilised the Langchain architecture to retain previous utterances in memory, enabling the preservation of context and maintaining a natural conversation flow. We utilised a dataset comprising ~ 1980 utterances from 180 participants from AphasiaBank, and the AI-reconstructed utterances achieved an accuracy of 80% using the GPT-4o model. We further investigated the impact of different speech errors on reconstruction accuracy to determine which errors affect the capability of LLMs to correct errors in impaired speech.

**Supplementary Information:**

The online version contains supplementary material available at 10.1038/s41598-025-24725-x.

## Introduction

Aphasia, a language impairment resulting from acquired brain injury or progressive disease, significantly impacts an individual’s ability to communicate effectively^1^. Aphasia results in various language impairments; most prominently, difficulty in finding the right words to express thoughts and challenges with sentence construction and comprehension. Aphasia can lead to fragmented speech, characterised by word errors, repetitions, and unfilled pauses, making communication challenging for those affected^2^. Aphasia not only affects regular day-to-day communication but can also lead to frustration, mood disturbance, social isolation and reduced quality of life^3^.

Given the surge in advanced Artificial Intelligence (AI) technologies, it is timely to explore the use of generative AI in assisting people with communication disabilities^4^. Specifically, the emergence of Large Language Models (LLMs) represents a transformative leap in natural language processing (NLP)^5^ with a multitude of possibilities for assistive communication. This applies especially to people with aphasia, whose communication is often incomplete, unrecognisable, or fragmented due to language processing impairments. LLMs exhibit numerous features that make them highly effective in generating text to fill in missing (masked) words in a given context^6^. Trained on vast datasets, LLMs possess a deep understanding of context, allowing them to predict and generate contextually appropriate responses seamlessly^7^. Therefore, LLMs’ capacity for language comprehension and generation positions them as invaluable tools for text completion in assistive communication.

### LLMs to compensate for impaired speech - Parallels between human sentence production and LLM-based sentence generation

**Fig. 1:**
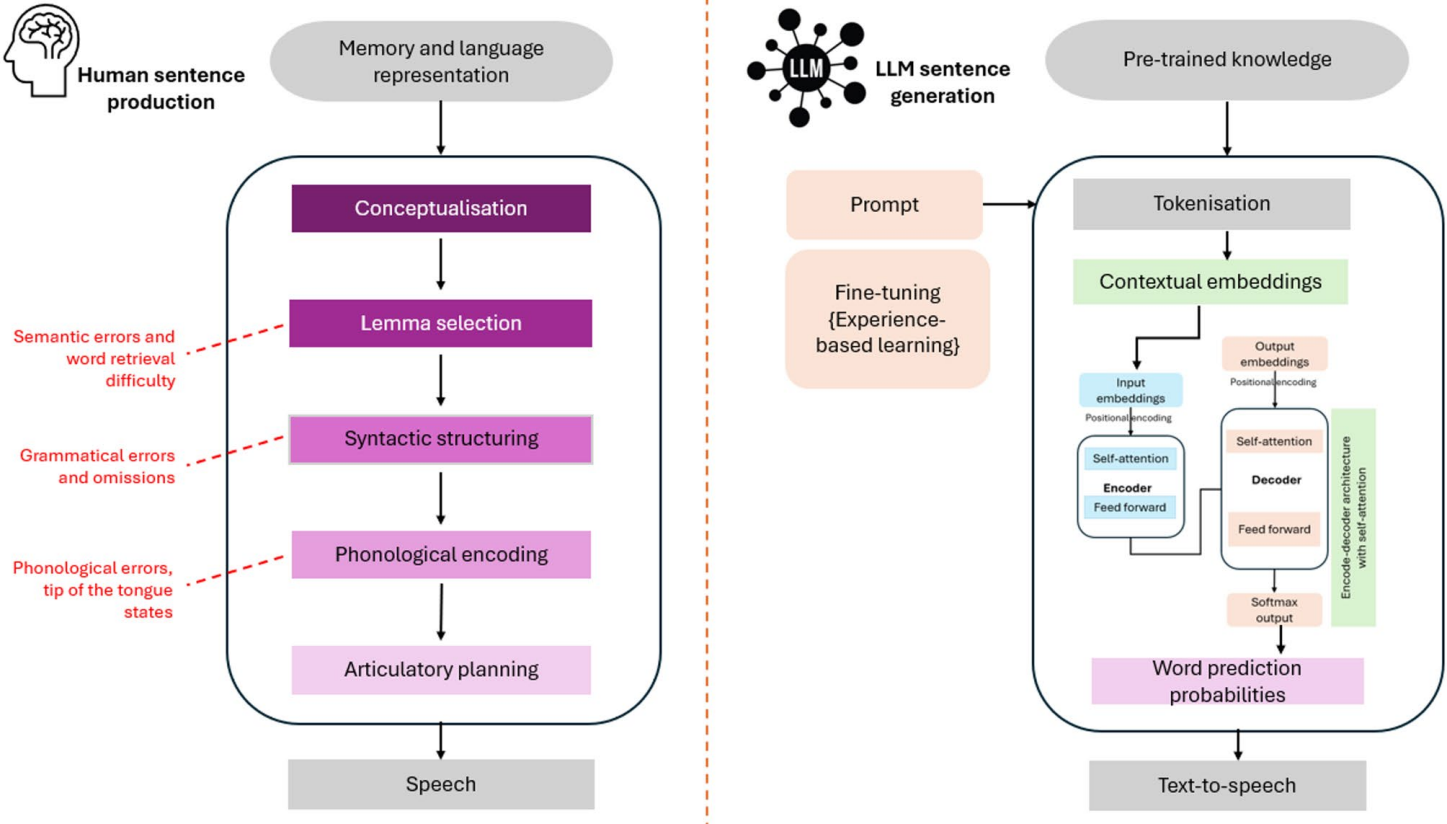
Parallels between human sentence production and LLM sentence generation. (Red dotted lines demonstrate errors made by people with Aphasia in these processes.)

The human sentence production system is a complex process involving the functionality of multiple neural networks^8^. While the sentence generation process in LLMs is not as intricate as the human language system, there are interesting similarities, indicating parallels between artificial intelligence and human cognitive functions. Figure 1 illustrates the speech and language production process and components of both humans’ and LLMs’, drawing from human speech production models^9^ and transformer-based language architectures^10^.

In human comprehension or response generation, the process begins with conceptualisation, where an idea or thought is formed that the individual wishes to express^11^. Following this, the human system engages in lexical selection, choosing specific words from its mental lexicon that best convey the intended meaning. Similar to the human system organising selected words into a syntactically sound structure, the LLMs rely on an encoder-decoder architecture (or similar transformer layers) to understand the relationships between tokens and structure them according to the rules of syntax^12^. This structuring is guided by focused attention and contextual processing in humans and LLMs. In humans, the system pays attention to the relevant context to maintain coherence in conversation, while LLMs use self-attention mechanisms to focus on the most pertinent parts of the input sequence, ensuring contextually appropriate predictions^5,13^.

People with aphasia experience difficulties in language production due to damage to specific networks in the brain critical for language processing^14^. This can lead to diverse language errors, such as selecting the wrong words or using parts of words (paraphasias), difficulty retrieving words, or constructing sentences with incorrect syntax (agrammatism). These errors can occur at various stages of speech production^15^. There are several subtypes of aphasia (e.g., Broca’s; Conduction; Wernicke’s) that represent differential impairments across the stages of speech and language processing. For example, during lexical selection, some individuals with aphasia may struggle to retrieve the appropriate word from competing entries in their lexicon, leading to the use of incorrect or related words (e.g., saying “dog” instead of “cat”). Word-finding difficulty may result in a compensation strategy whereby multiple words are used to describe the target concept, a phenomenon known as circumlocution. During the syntactic structuring phase, some individuals with aphasia may produce sentences with incorrect grammar or missing elements, making communication unclear.

During phonological processing in word production, the correct sequence of sounds is selected, arranged and then articulated, relying on coordination across brain regions^16^. Impairments can lead to phonological errors, most common in Broca’s or Conduction aphasia, manifesting as substitutions (e.g., “chat” for “cat”), omissions (e.g., “comicate” for “complicate”), additions (e.g., “clat” for “cat”), or transpositions (e.g., “tat” for “cat”). In these scenarios, LLMs could potentially assist by using surrounding context to correct such errors, for example, suggesting “table” when “bable” is incorrectly said. The LLM’s attention mechanisms focus on sentence structure and context to aid this process of correction, making LLMs powerful tools for language correction.

### Current research using LLMs in aphasia

Given these capabilities of LLMs, contemporary research has explored the potential of their use in aphasia research. Integrating LLMs into a natural language processing pipeline could significantly improve the modelling of language impairments like aphasia, leading to more refined and effective diagnostic tools^17^. Additionally, LLMs could be fine-tuned on personalised linguistic patterns, adapting to individual communication needs and supporting multi-modal integration (e.g., speech-to-text, gestures) to enable functional communication. Their scalability and real-time processing have the potential of making them valuable tools for augmenting traditional speech therapy practices and improving assistive communication technologies.

A recent study presented the clinical efficacy of using LLMs to measure sentence information and extract language features in conversations of people with aphasia^17^ with the view to automating aphasia severity assessments. However, applying LLMs as an assistive or compensatory agent needs further investigation. In a study by Salem et al.^18^, the LLM BigBird was fine-tuned to predict targets of paraphasias from story retellings by individuals with aphasia, achieving 66.8% for the top 5 predicted words. The study focused on a constrained task (retelling the Cinderella story) and recommended that future work should apply the method to more naturalistic speech tasks for broader applicability. Purohit et al.^19^ explored ChatGPT’s ability to identify intended words in circumlocution produced by individuals with aphasia. However, the study used only 12 sentence samples, indicating the need for larger datasets and refined prompt engineering to enhance communication support in future research. Valencia et al.^20^ investigated LLMs in augmentative and alternative communication (AAC) devices for individuals with communication challenges, largely anarthric speech (anarthria is a motor speech disorder whereby muscle weakness and/or incoordination impairs clear articulation or prevents articulation entirely). While AAC users appreciated AI-generated phrases for reducing effort and communication time, they noted the lack of personalisation limited the acceptability. The exploratory Valencia et al. study used a small sample size, with 12 participants generating only 5–10 responses.

Overall, these studies demonstrate the promise of LLMs in aiding communication for individuals with aphasia but also highlight the need for further deeper exploration, larger datasets, and improved personalisation to ensure effectiveness in diverse, real-world settings.

Given this background, investigating the design and development of an intelligent system that can enhance communication for people with aphasia with the use of emerging technologies^21^ is important and timely. Our investigation identified gaps in the current literature, where research on assistive communication for aphasia has not kept pace with the exponential growth of generative AI and, as such, identified potential opportunities for technological advancement. Consequently, we propose an intelligent conversation system with robust training and evaluation methodologies. Importantly, we demonstrate the system’s efficacy for more naturalistic conversations, evaluating its performance against different speech errors produced by people with aphasia, making it more applicable and usable for this cohort.

## Methods

### Solution overview

The proposed solution is an intelligent conversational agent designed to reconstruct fragmented language for individuals with aphasia using LLMs and the Langchain architecture as building blocks. In contrast to recent research in using LLMs for aphasia language correction, we designed a framework to cater to a conversation flow with (1) more natural conversations rather than catering to a specific restricted topic and (2) in-built memory to acquire and preserve context from previous conversations from the participant. From a computational perspective, we showcase the development and evaluation of an interactive, real-time dialogue system using Langchain and GPT-4o as building blocks, capable of transforming fragmented utterances into corrected utterances with preserved memory of prior conversations.

**Fig. 2:**
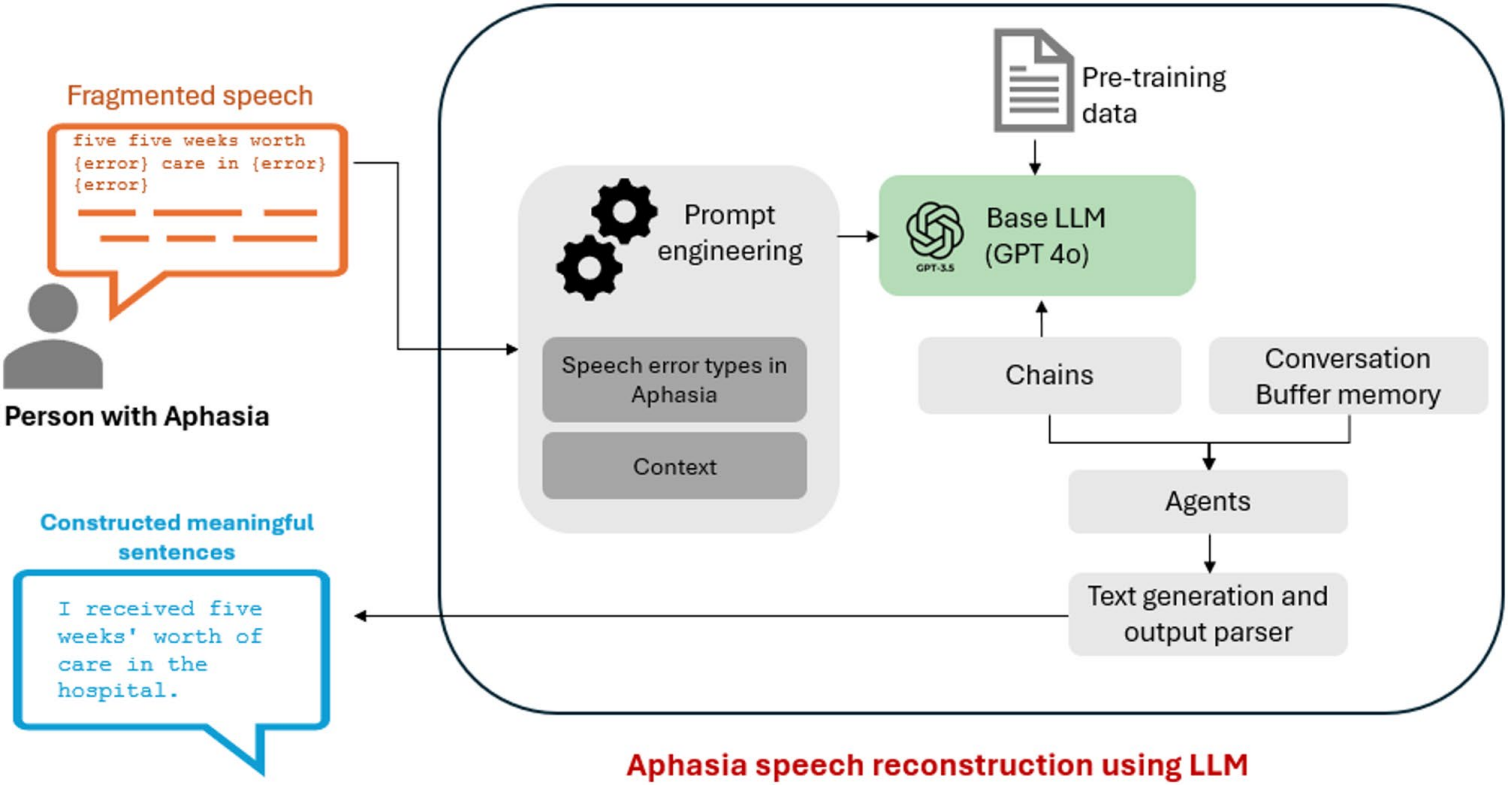
The proposed solution for a conversation assistant system.

Moreover, we utilised prompt augmentation and few-shot learning to train the LLM with different language errors in aphasic speech. This process enables the LLM to better comprehend the language errors of individuals with aphasia, thereby generating more accurate outputs than standard LLMs. Figure 2 shows the high-level overview of the proposed system.

### Data description

The AphasiaBank dataset stands as a significant and comprehensive resource in the field of aphasia research, providing a rich collection of language samples from individuals affected by aphasia^22^. This database has been curated with meticulous attention to detail to capture variations in aphasic speech^23^. AphasiaBank consists of interviews between patients with aphasia and speech pathologists. Each interview contains a segment where the speech pathologist asks general questions about the stroke, recovery, and essential incidents to assess speech quality, followed by formal language assessment tasks. Most prior AI studies have focused on such formal and structured language tasks, which do not appraise the potential of LLM application in a natural conversation setting. Targeting this gap in research, we have focused on the first segment of the AphasiaBank protocol interview, where the speech pathologist asks open-ended questions about stroke, recovery, and their experience. We used data from 180 patients based on data availability in the AphasiaBank repository. The selected 180 transcripts contained full transcriptions from people with aphasia, resulting in 1982 utterances for evaluation.

### Prompt augmentation via in-context few-shot learning

Just as humans refine speech through experience, LLMs are trained on large datasets to learn language patterns, grammar, and context and can be further tailored for specific tasks. In this study, we applied this learning approach by instructing the LLM with a team member speech pathologist’s understanding of language errors, aiming to assist individuals with aphasia by predicting and correcting errors.

In this study, we employed *in-context few-shot learning* to guide GPT-4o in reconstructing fragmented speech from individuals with aphasia. Few-shot learning refers to the technique of learning the underlying pattern in the data just from a few training samples^24^. Contrary to conventional fine-tuning, no model weights were updated. Instead, the model was instructed using carefully designed prompts that included a small number of representative examples, allowing the model to generalise patterns and correct errors at inference time^25^.

Five representative prompt examples were constructed for each language error category, including phonological substitutions, semantic errors (related and unrelated), neologisms (known and unknown targets), and morphological errors. Each example included an erroneous utterance and its corrected reference. These in-context demonstrations were prepended to the prompt provided to the LLM during inference.

The model was not trained using PEFT (Parameter-Efficient Fine-Tuning), LoRA (Low-Rank Adaptation), or any weight-updating technique. The generative responses were solely influenced by prompt design and conversation memory using Langchain.

The dataset was partitioned as follows:

- Few-shot prompt examples (manual input): 5 examples per error type, excluded from evaluation data to prevent test leakage.
- Test set: 1,982 utterances from 180 participants in the AphasiaBank dataset.

Prompt-based inference was conducted using OpenAI’s GPT-4o model with parameters configured to balance creativity and coherence. Specifically, we set the temperature to 0.7 to allow for moderately diverse outputs, while maintaining a top-p value of 1.0 to include the full probability distribution during token sampling. The maximum token limit for each generated response was capped at 512 tokens to ensure completeness within a single conversational turn. Additionally, the conversation memory buffer in Langchain was configured to retain six utterances (equivalent to three user-therapist exchanges), enabling the model to preserve contextual flow and generate more contextually relevant reconstructions.

**Fig. 3:**
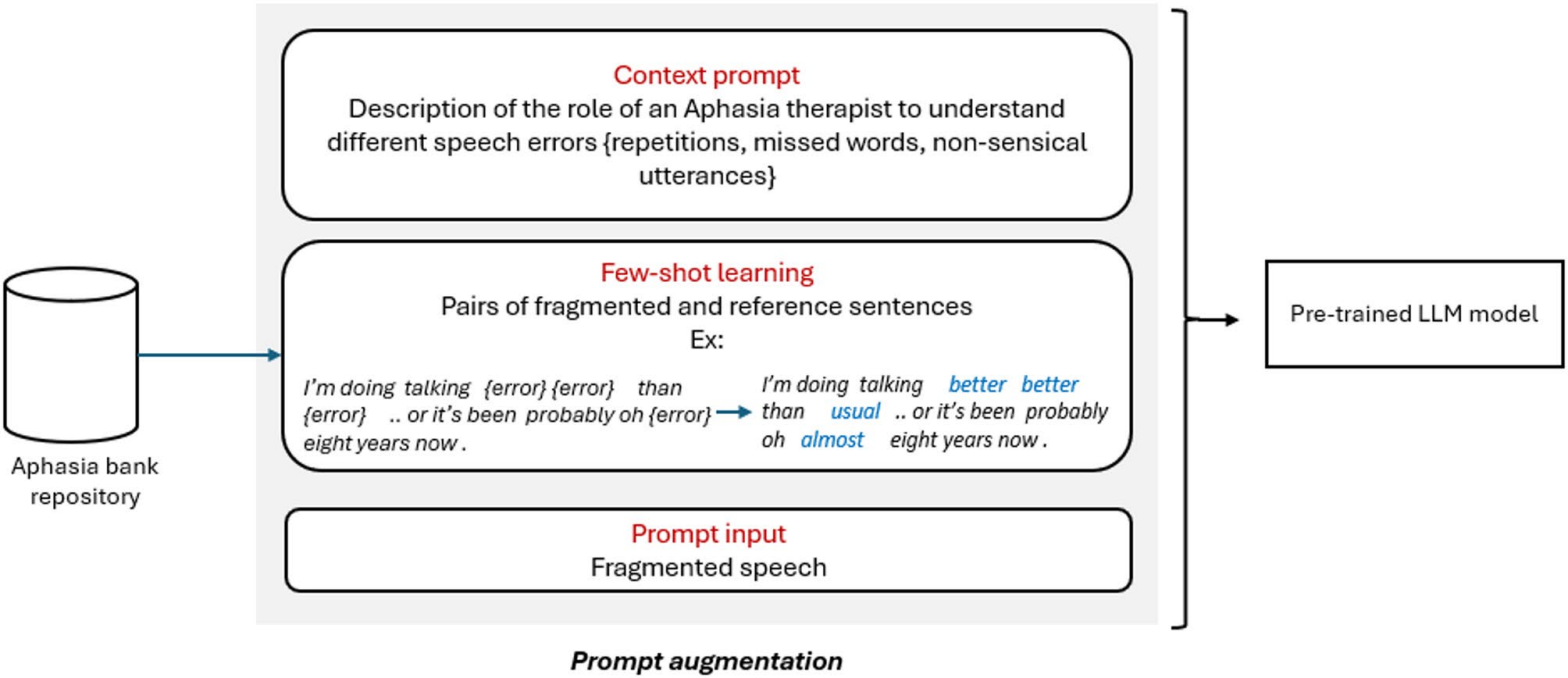
Prompt augmentation process.

Figure 3 illustrates the prompt augmentation process.

This targeted training helps improve the model’s generalization capabilities, making it more reliable and effective in real-world scenarios where diverse language errors may occur.

### LLM and Langchain integration

The Langchain architecture stands out as an important choice for dialogue systems, as it is tailored to support sequential conversation chains to simulate the flow of human dialogue^26^. The Langchain architecture employs “chains,” sequences of interconnected components for tasks like question answering and dialogue generation. Each chain is encapsulated into an “agent,” streamlining user interaction with the LLM. This empowers conversational systems to comprehend and generate contextually relevant responses^27^.

Moreover, the Langchain architecture supports different types of memory that can retain the history of the conversation. The memory parameters can be set to adjust based on the length of interactions dynamically. Given the sequential nature of the interview data, we used conversation buffer memory as this memory instance retains the history of previous utterances, allowing the model to consider both the immediate and broader context of the conversation. The size of this buffer, represented by the memory parameter *“k”*, was dynamically adjusted based on the length of each conversation to understand the interview context. We present this as a novel addition to discourse analysis in Aphasia, as it enables the LLM to refer to the recent past as well as conversation in general. Referring to recent past utterances would provide more details about the focus of the conversation, thereby resulting in a more context-aware prediction. In this research we used a recent memory of 3 multi-turns that resulted in consideration of 6 utterances by the therapist and patient.

The text generation was based on BART, OpenAI’s GPT 3.5-turbo and GPT-4o models for reconstructing the fragmented speech. Using such encoder-decoder architecture and attention mechanisms, LLMs can address language errors by focusing on relevant sentence parts and reorganising them into grammatically correct, contextually appropriate forms, helping reconstruct disrupted sentences for clearer communication^28^.

### Evaluation

**Fig. 4:**
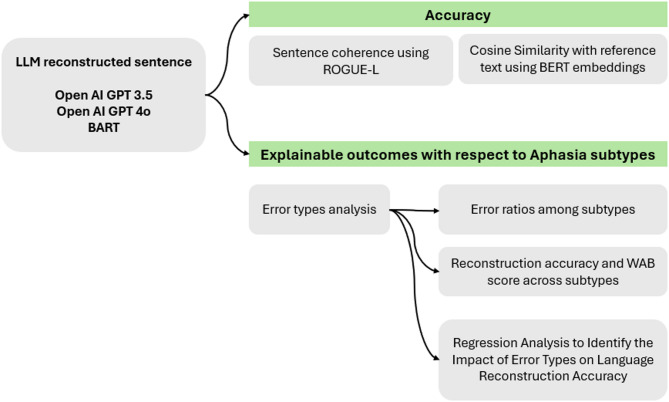
Evaluation process.

A comprehensive, multi-faceted evaluation was conducted to assess the accuracy of LLM-generated sentence reconstructions, alongside an in-depth analysis of aphasia-related speech error types as shown in Fig. 4. The study compared error patterns across different aphasia subtypes and severity levels to identify which error types are most challenging for LLMs and which subtypes experience the greatest penalties in reconstruction accuracy. Additionally, a regression analysis was performed to determine which specific speech errors most significantly impact the performance of LLM-based sentence reconstruction.

#### Comparison of annotated vs. machine-generated responses

To ensure the reliability of the model, BERT embeddings were utilised to generate dense, informative word embeddings and capture contextual information in sentence representations^29^. Unlike sparse vectors in one-hot encoding, BERT’s dense vectors store meaningful values in each element, enhancing their ability to encode nuanced semantic information. Embeddings were generated for reference and used to reconstruct sentences using the Sentence Transformer library in Python^30^. Cosine similarity was then applied to evaluate the similarity between these embeddings, providing a robust measure for assessing the quality of reconstructed sentences. Cosine similarity, a metric measuring the similarity between two vectors representing the sentences, was used for this evaluation^31^. This similarity score aimed to assess how closely the predicted word (A) aligned with the intended meaning and context (B), providing insights into the prediction’s effectiveness. A higher similarity score indicates that the two instances are semantically similar.

Cosine Similarity (A, B) = (A · B) / (||A|| * ||B||).

In LLMs, ROUGE (Recall-Oriented Understudy for Gisting Evaluation) measures play a crucial role in evaluating the quality and coherence of automatically generated content^32^. ROUGE-L is a metric used to evaluate the quality of machine-generated text, particularly in the context of machine translation. The “L” in ROUGE-L stands for Longest Common Subsequence (LCS). Instead of focusing on n-gram overlap, as seen in ROUGE-N measures, ROUGE-L measures the length of the longest common subsequence between the generated text and the reference text. It is computed using the precision and recall of the matching n-grams in the reference and generated text.

ROUGE-L F1-score = 2 * (precision * recall) / (precision + recall).

#### Analysis of speech features

Speech errors in the Aphasia Bank dataset have been systematically and manually coded in the transcripts using the CLAN (Computerized Language ANalysis) software^33^, which provides a robust framework for analysing language and speech data. Each error type—phonological, semantic, neologistic, morphological, and dysfluent—has been marked with specific codes (e.g., *p* for phonological, *s* for semantic) to ensure consistent and detailed documentation. CLAN enables efficient tagging of these categories, allowing for precise differentiation between error types and facilitating analysis of error patterns across participants.

This coding approach supports the identification of underlying linguistic challenges and provides a structured basis for further quantitative analysis.

**Table:** 

Phonological Errors [*p*]*	p_w (Word Substitution)	Substitution with a real word that resembles the target phonologically, e.g., boater for butter.
	p_n (Non-word Substitution)	Substitution with a non-word that resembles the target, e.g., buther for butter.
	p_m (Metathesis)	Reordering of sounds within the word, e.g., stisserz for sisters.
Semantic Errors [s]*	s_r (Related Word, Target Known)	Use of a semantically related word, e.g., mother for father.
	s_ur (Unrelated Word, Target Known)	Use of an unrelated word with a known target, e.g., comb for umbrella.
	s_uk (Word, Unknown Target)	Use of a word without a clear target, e.g., “I go wolf.”
	s_per (Perseveration)	Repetition of a previously used word or phrase, e.g., “he kicked the ball through the ball.”
Neologisms [n]*	n_k (Neologism, Known Target)	Creation of a novel word with a known target, does not meet phonological error criteria.
	n_uk (Neologism, Unknown Target)	Creation of a novel word without a clear target.
Morphological Errors [m]*	m_a (All Morphological Errors)	Includes any deviation from the correct morphological form, e.g., incorrect verb conjugations, affix substitutions.
Dysfluencies [d]*	d_sw (Within-word Dysfluency)	Dysfluency within a word, e.g., insuhside for inside.

### ​Output parser

The output parser module processes the output to construct it in a readable way to the user. During this process, standard answering templates of the GPT-4o model were removed, and only the answer was retained.

## Experiments and results

### Exploring natural conversation data of aphasia bank

The data selection for this study consisted of extracting the initial open ended questions component, consisting of free form conversations from the Aphasia Bank dataset, as these questions represent more general conversations. Other parts of the conversations which are more structured and focused on specifics may be overly context dependent (e.g., narrating the Cinderella story), which can hinder the prediction capability of the proposed system in naturalistic daily conversations.

The scripts were processed to isolate question-and-answer pairs between therapists and patients. During this phase, the CLAN software annotated incorrect words and was used for masking as {error} instances. For each patient utterance, both the erroneous and reference sentences were segmented. The primary objective was to utilise the erroneous sentences for text generation and the reference sentences (labelled data) as the reference texts for evaluation. A total of 1982 utterances from 180 patients underwent this coding procedure as part of the data preparation phase. Examples are shown below for clarity in Table 1.

**Table 1:** Examples of fragmented speech and reference speech corrected via annotation in aphasiabank corpus.

Fragmented speech	Reference speech via annotation by CLAN
*I’m doing talking {error} {better} than {error} . or it’s been probably oh {error} eight years now .*	*I’m doing talking better better than usual . or it’s been probably oh almost eight years now .*
*I I read {error} . and and then I can make {error} . then and follow up with a a a a comment . and so I’m {error} well .*	*I I read paragraph . and and then I can make sentence . then and follow up with a a a a comment . and so I’m doing well .*
*depending on the day it’s pretty {error} it’s pretty {error} . and I it’s not so {error} when I’m {error} or I don’t feel good . so {error}*	*depending on the day it’s pretty good it’s pretty good . and I it’s not so good when I’m tired or I don’t feel good . so*

### Sentence reconstruction using LLMs

To assess the effectiveness of sentence reconstruction, we evaluated the outputs from GPT-3.5-turbo and GPT-4o using multiple metrics. The primary measure of reconstruction accuracy was cosine similarity, which evaluates semantic alignment between the model-generated sentence and the reference sentence using BERT embeddings. This approach reflects the model’s ability to generate contextually appropriate responses, a critical consideration in conversational settings involving aphasia. Before diving into LLM models, we compared the performance of baseline heuristic models for correcting erroneous sentences with earlier LLM models, specifically BART, to assess their performance.

We utilised a simple baseline based on a sequence tagging grammar correction approach, an earlier LLM model and improved GPT-3.5 and GPT-4o models for comparison. As the simple baseline model, we included a simple heuristic corrector (hand-coded rules for articles, subject–verb agreement, and edit-distance normalisation) and a lightweight sequence-tagging baseline to evaluate how erroneous utterances are corrected by simple baseline models. A paired t-test confirmed that GPT-4o significantly outperformed this heuristic corrector model (t = 4.68, *p* < .01). Paired bootstrap resampling (*n* = 10,000) yielded a 95% confidence interval of [0.054, 0.131], further supporting the statistical robustness of the performance gain.

Next, as the early LLM-based baseline, we selected BART for its broader applicability and interpretability. However, GPT-4o demonstrated a clear advantage over BART in generating contextually appropriate reconstructions. A paired t-test between cosine similarity scores confirmed that this improvement was statistically significant (t = 7.34, *p* < 1.6 × 10^−12^). To further validate this finding, we applied paired bootstrap resampling (10,000 iterations), which produced a 95% confidence interval of [0.077, 0.134] for the mean difference in performance.

Given the limitations of BART in tokens and memory capabilities and inherent limitations in heuristic models in generating sentences, these findings reinforce the effectiveness of GPT-4o over earlier LLMs and support its potential for real-world assistive applications in aphasia communication. Although prior work by Salem et al. (2023) employed BigBird for aphasia-related reconstruction, we opted not to include BigBird in our study. The model showed relatively low performance (66.8% top-5 prediction accuracy) and was evaluated in a narrow domain (storybook retelling), which does not align with our focus on naturalistic conversational speech.

We evaluated the accuracy of the LLM generated and the actual sentences which resulted in a 77.45% average similarity using the GPT-3.5 turbo model and 80.00% using the GPT-4o model. A paired-samples t-test indicated a significant advantage for GPT-4o, *t*(1981) = 22.42, *p* = 2.11 × 10^−99^, with a 95% CI for the paired difference of [0.0236, 0.0299]. The corresponding paired effect size was Cohen’s *d*_n_ = 0.50 (medium). A Wilcoxon signed-rank test corroborated this result (two-sided *p* = 5.29 × 10^−127^; *r* = .54), confirming that GPT-4o outperforms GPT-3.5 by a moderate-to-large margin. Both a robust non-parametric test and a standard parametric effect size indicate that GPT-4o outperforms GPT-3.5, with a moderate-to-large advantage. Notably, newer-generation LLMs such as GPT-4o exhibit superior capabilities in contextual understanding and prompt responsiveness, enabling them to interpret fragmented or ambiguous inputs more accurately and generate more meaningful, contextually aligned outputs. In addition to cosine similarity, we report ROUGE-L, which measures sequence overlap based on the longest common subsequence between predicted and reference texts. While ROUGE-L does not capture contextual meaning, it offers a supplementary view of structural coherence. The average measure of ROUGE-L using GPT-3.5-turbo was 71.6%, and GPT-4o was 76.42%, indicating better coherence and consistency in the advanced model.

Table 2 presents both cosine similarity and ROUGE-L scores for GPT-3.5 and GPT-4o. Table 3 compares these results with prior approaches. Note that reconstruction accuracy in these tables specifically refers to cosine similarity, which we use as our primary accuracy metric given its relevance to semantic understanding. For evaluation, we rescale cosine similarity values from [0,1] to [0%,100%] by multiplying by 100. Note that in our experimental pairs, cosine similarities are empirically observed to lie in [0,1] due to the nature of the semantic matching task.

**Table 2:** Evaluation of the outcomes.

Accuracy measured against the reference text	Model: GPT 3.5-turbo	Model: GPT 4o
Cosine similarity	77.45%	80.00%
ROUGE-L	71.60%	76.42%

**Table 3:** Comparison with other approaches.

Study	Accuracy	Number of input samples	Model	Context
Salem et al., 2023	66.8%	2,489	BigBird	Cinderella storytelling
Purohit et al.,2023	91.6%	12	ChatGPT	Storytelling exercise
**Proposed approach**	**77.45%**	**1**,**982**	**GPT 3.5-turbo + Langchain**	**Natural conversations**
	**80%**	**1**,**982**	**GPT 4o + Langchain**	**Natural conversations**

The more recent and advanced model GPT-4o showcased improved performance. The following sample utterances demonstrate the ability to reconstruct language (Table 4).

**Table 4:** Outcomes: examples of reference and LLM-constructed speech.

Fragmented speech	CLAN-annotated speech (reference)	LLM Reconstructed sentence	Similarity score
*I’m doing talking {error} {error} than {error} . or it’s been probably oh {error} almost eight years now .*	*I’m doing talking better better than usual . or it’s been probably oh almost almost eight years now .*	*I’m doing talking much better than before or it’s been probably oh gosh almost eight years now.*	*0.838525*
*I I read {error} . and and then I can make {error} . then > and follow up with a a a a comment . and so I’m {error} well .*	*I I read paragraph . and and then I can make sentence . then > and follow up with a a a a comment . and so I’m doing well .*	*I read books and then I can make notes and follow up with a comment*,* so I’m doing well.*	*0.757493*
*depending on the day it’s pretty {error} it’s pretty {error} . and I it’s not so {error} when I’m tired or I don’t feel good . so {error}*	*depending on the day it’s pretty good it’s pretty good . and I it’s not so good when I’m tired or I don’t feel good . so*	*depending on the day it’s pretty good*,* it’s pretty clear and I it’s not so good when I’m tired or I don’t feel good.*	*0.95751*

While cosine similarity and ROUGE-L offer useful quantitative metrics for evaluating model output, they do not always capture clinical adequacy or semantic correctness. For instance, phrases like “I am hungry” and “I am not hungry” may receive high similarity scores despite conveying opposite meanings, a critical issue in contexts like healthcare, where nuanced language matters (Table 5).

**Table 5:** Possible misinterpretations in data.

Fragmented Input	Reference (CLAN)	LLM Output	Cosine Similarity	Clinical Evaluation
I feel {error} now	I feel tired now	I feel fine now	0.89	Misrepresents intent
I {error} go to {error}	I want to go to school	I love to go to school	0.86	Ambiguity in intent
I’m {error} to eat	I’m ready to eat	I’m ready to eat	0.92	Correct and contextually appropriate
He {error} the ball again	He kicked the ball again	He hit the ball again	0.87	Acceptable paraphrase
I don’t feel {error}	I don’t feel good	I feel good	0.88	Negation reversed

To mitigate this limitation, our approach integrates conversational memory using Langchain’s buffer memory architecture. By preserving previous therapist-patient exchanges, the model can reference prior utterances, maintaining semantic continuity and improving its ability to disambiguate short or fragmented input. This memory mechanism strengthens the model’s capacity to generate contextually accurate and clinically useful reconstructions, rather than relying solely on surface-level similarity.

As an additional validation step, we tested a sample of 40 LLM-generated responses with two certified speech pathologists to gain insights into the clinical relevance of the responses. The following table presents their results in terms of Correctness, grammaticality, and Semantic fidelity, using a 5-point Likert scale (1 = poor, 5 = excellent) (Table 6).

**Table 6:** Evaluation of outputs with speech Language pathologists.

Dimension	Reviewer 1	Reviewer 2
Correctness	4.38	4.36
Grammaticality	4.36	3.93
Semantic Fidelity	4.17	3.90

These results indicate a generally high level of satisfaction with the LLM-generated reconstructions across all three clinical dimensions. Ratings for correctness and grammaticality were particularly strong, averaging above 4.0 for both reviewers, suggesting that the outputs were typically well-formed and aligned with clinical expectations.

Scores for semantic fidelity were slightly lower, reflecting occasional challenges in preserving the exact intended meaning of the original utterances an important consideration in aphasia communication. Inter-rater reliability was assessed using Cohen’s kappa, yielding moderate agreement across criteria: κ = 0.31 for correctness, κ = 0.33 for grammaticality, and κ = 0.30 for semantic fidelity. This variation also supports our decision to include conversational memory in the model architecture, enhancing contextual accuracy.

These values indicate fair agreement, which is expected in clinical judgment tasks involving nuanced and subjective interpretation. The variability underscores the inherent complexity in assessing language reconstructions for aphasia and the need for continued refinement of rating protocols.

#### Evaluation of LLM capabilities across different Language errors in aphasia

Although LLMs are equipped with standard language generation capabilities, a more in-depth analysis of where and when LLMs fail to reconstruct speech is required when dealing with impaired speech, as aphasic speech deviates considerably from natural language. Therefore, further investigation was carried out in this research as a deeper investigation to analyse how LLMs handle diverse types of speech errors present in different aphasia subtypes.

**Fig. 5:**
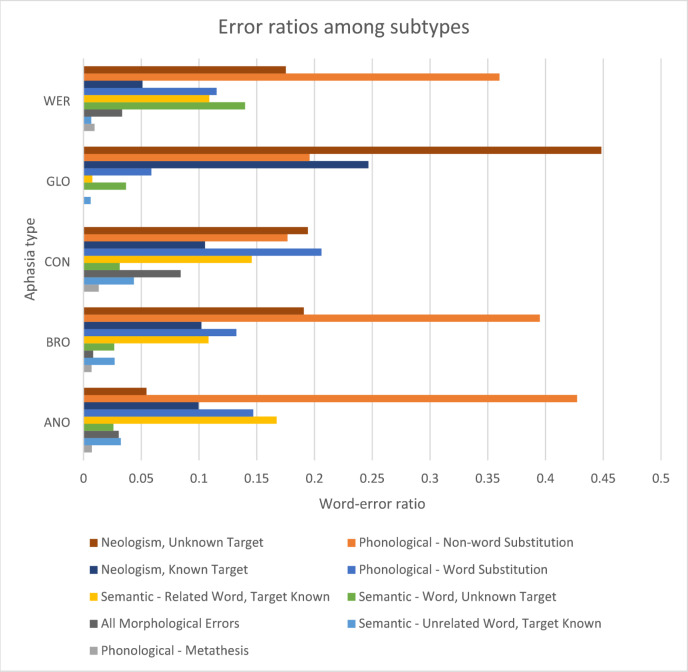
Different error types across aphasia subtypes.

An exploratory analysis was conducted to identify the prevalence of different errors across aphasia subtypes, providing insight into the existence of specific linguistic impairments in aphasia as shown in Fig. 5. The bar chart presents the word-to-error ratios calculated considering the number of errors across all the words. The error profiles of different aphasia subtypes provide insight into what errors impact the LLM’s prediction accuracies.

Global aphasia (GLO) exhibits the highest proportion of Neologism - Unknown Target errors, indicating a significant difficulty in producing recognisable words. Similarly, Neologism - Known Target errors are most prominent in Broca’s aphasia (BRO), suggesting that word formation remains highly impaired even when the target word is known. Wernicke’s aphasia also had higher neologism errors and phonological non-word substitutions indicating more irrelevant words in conversations.

Semantic errors, such as Semantic - Related Word, Target Known, appear more frequently in Broca’s aphasia and Global aphasia, indicating that individuals with these types struggle with selecting the correct word from a semantically related set. Phonological - Non-word Substitution errors are more pronounced in Global and Broca’s aphasia, aligning with the observation that phonological processing difficulties are a key characteristic of these types.

Conduction aphasia (CON) and anomic aphasia (ANO) display lower overall error rates compared to Global and Broca’s aphasia, with errors being more evenly distributed among phonological and semantic categories. Within-word dysfluency is relatively minor across all aphasia types.

These differences in speech errors across aphasia types warrant further exploration to determine how they may impact the accuracy of the LLMs, and identify how the LLMs can be directed, outputs tailored to address the specific needs and characteristics of each aphasia type.

##### Exploring aphasia type/severity and language reconstruction accuracy.

The reconstruction accuracy was compared across the severity of Aphasia, measured by the Western Aphasia Battery - Aphasia Quotient (WAB-AQ)^34^. This standardised measure is used to assess the severity and classification of aphasia. It provides a composite score derived from key language domains, including spontaneous speech, comprehension, repetition, and naming, offering a quantitative index of aphasia severity. A lower WAB AQ score indicates more severe aphasia.

Table 5 displays the average accuracy of the LLM in reconstruction for each aphasia subtype. The original dataset consisted of 180 participants. However, for the purpose of regression analysis and reconstruction accuracy by aphasia subtype, we excluded 12 participants who belonged to underrepresented categories (i.e., subtypes with only 1–2 individuals. Therefore, Table 5 reports reconstruction accuracy (mean cosine similarity) only for the five most prominent aphasia subtypes.

Overall, the LLM accuracy was highest for Conduction aphasia, indicating that language from individuals with this subtype are clearer for LLMs than other subtypes. Conversely, the lowest accuracy was observed for Global aphasia, which aligns with its low mean overall aphasia severity (WAB-AQ = 24.5), suggesting that more severe language deficits hinder LLM reconstruction performance. The LLM showed moderate accuracy for Anomic and Broca’s aphasia, while Wernicke’s aphasia fell slightly below them. This pattern highlights the relationship between aphasia severity and type with the effectiveness of language model reconstruction.

There was a significant weak-moderate positive correlation between WAB-AQ score and reconstruction accuracy (*r* = .309, *p* < .05), suggesting that the LLM shows more considerable challenges in language reconstruction for individuals with more severe aphasia. At the same time, those with milder impairments may produce errors that are more systematic and easier for the model to correct. Table 7 presents the accuracy levels and mean WAB AQ score across aphasia types.

**Table 7:** Reconstruction accuracy and WAB score across aphasia subtypes.

Aphasia subtype	Reconstruction accuracy	MEAN WAB Score
CON	0.888	66.633
ANO	0.860	87.625
BRO	0.819	61.578
WER	0.816	54.267
GLO	0.747	24.478

The model showed the highest reconstruction accuracy (0.888) for Conduction Aphasia (CON) despite a moderate mean WAB AQ (66.633). Figure 4 shows that people with Conduction aphasia have low levels of unknown targets (Neologism; Semantic) and Phonological Substitution errors, and this may make it easier for the LLM to predict the target and correct the error.

The model also showed high reconstruction accuracy (0.860) in Anomic Aphasia (ANO) alongside the highest mean WAB-AQ score (87.625). According to Fig. 4, the Anomic aphasia subtype also demonstrated lower levels of unknown target errors, which makes it easier for the LLM to correct, thus providing a higher accuracy.

The model had similar reconstruction accuracies for Broca’s Aphasia (BRO) and Wernicke’s Aphasia (WER) (0.819 and 0.816, respectively). The Fig. 4 shows broadly similar error profiles for these subtypes, which could have led to comparable reconstruction outcomes.

Global Aphasia (GLO) had the lowest reconstruction accuracy (0.747), though this was still moderately high, and was the most severe aphasia (WAB-AQ 24.478), indicating that severe language impairments significantly hinder the LLM’s ability to reconstruct speech. The widespread deficits in word and sentence production and, high levels of neologism (both known and unknown) as shown in Fig. 4in global aphasia likely make it more difficult for the model to generate meaningful reconstructions.

These findings highlight the varying challenges posed by different aphasia types and encourage the exploration of the impact of different speech errors on LLM performance.

##### Regression analysis to identify the impact of error types on language reconstruction accuracy

We first assessed the Ordinary Least Squares (OLS) residuals and found that they substantially violated the normality assumption (Shapiro–Wilk W = 0.846, *p* < .001). Because our outcome (reconstruction accuracy) is a bounded proportion and the Gaussian assumption was untenable, we instead fitted a Generalised Linear Model. This approach helps determine which error types significantly impact the model’s ability to reconstruct speech by analysing their individual contributions while controlling for other factors. By examining the coefficients and statistical significance of each predictor, we can identify whether specific errors, such as phonological, semantic, neologistic, or morphological errors, have a more substantial influence on accuracy. This insight is crucial for refining reconstruction models, as it highlights which linguistic challenges require more targeted improvements in computational approaches.

Type III Wald χ^2^ tests from this GLM showed that several error-type predictors made significant contributions to reconstruction accuracy, as shown in Table 8.

**Table 8:** Generalised linear model outcomes.

Predictor	Wald χ^2^	*p*-value
Phonological – Word Substitution	49.72	< 0.001
Phonological – Non-word Substitution	73.92	< 0.001
Semantic – Related Word, Target Known	48.97	< 0.001
Semantic – Unrelated Word, Target Known	9.88	0.002
Neologism, Known Target	9.84	0.007
Neologism, Unknown Target	39.84	< 0.001

The analysis revealed that several error-type features exert a significant influence on reconstruction accuracy. In particular, both phonological error categories, word substitutions (χ^2^₍_2_₎ = 49.72, *p* < .001) and non‐word substitutions (χ^2^₍_12_₎ = 73.92, *p* < .001) were highly predictive. Likewise, semantic errors, whether substituting related words when the target is known (χ^2^₍_4_₎ = 48.97, *p* < .001) or substituting unrelated words (χ^2^₍₁₎ = 9.88, *p* = .002), significantly impacted performance. Finally, neologisms both with known targets (χ^2^₍₂₎ = 9.84, *p* = .007) and unknown targets (χ^2^₍_2_₎ = 39.84, *p* < .001) also showed reliable effects. These results indicate that phonological, semantic, and neologistic disruptions each play a key role in determining how accurately the model can reconstruct aphasic speech. By contrast, All Morphological Errors (χ^2^_(1)_ = 0.14, *p* = .71) and Within-word Dysfluency (χ^2^_(1)_ = 2.97, *p* = .085) did not reach significance.

Errors related to ‘Phonological substitutions’ (word substitutions (χ^2^₍_2_₎ = 49.72, p < .001) and non-word substitutions (χ^2^₍_12_₎ = 73.92, p < .001)) have a notable effect on reconstruction accuracy. Phonological errors are known to be challenging even in therapy settings by speech pathologists due to their complexity^35^. Since the interviews are transcribed manually, phonological errors appear akin to spelling mistakes to language models. Phonological errors often result in non-existent words that do not appear in standard LLM training datasets. Unlike typical misspellings that resemble real words (e.g., “teh” instead of “the”), non-word substitutions (e.g., “busher” for “butter”) may not have a clear target, making it difficult for the model to infer the intended word. LLMs primarily rely on text-based patterns rather than phonetic reasoning. Unlike humans, who use auditory and phonetic cues to recognise mispronunciations, LLMs process only textual data. This can result in errors when reconstructing words that deviate significantly from standard spellings and typical misspellings due to phonological substitutions. As a result, LLMs might lack the ability to accurately handle speech-derived spelling mistakes that do not follow conventional misspelling patterns.

Neologisms, both with known targets (χ^2^₍₂₎ = 9.84, *p* = .007) and unknown targets (χ^2^₍_2_₎ = 39.84, *p* < .001) also showed reliable effects. Neologisms are types of word-finding difficulties and create vague and often circumlocutory expressions that lack specificity. This lack of precise context for LLMs complicates word prediction, as the model relies on contextual clarity to accurately infer and replace missing or misused words. The model struggles to identify exact substitutions in neologisms and instances of semantic errors with unknown targets, resulting in lower accuracy. This highlights the limitation of LLMs in situations where context is insufficient or ambiguous.

A high Wald χ^2^ for Semantic–Related Word, Target Known (χ^2^₍_4_₎ = 48.97, *p* < .001) indicates that when individuals with aphasia substitute a semantically related word (e.g., saying “mother” instead of “father”), this error type has one of the strongest impacts on the LLM’s reconstruction accuracy. Clinically, such paraphasic errors arise from a retrieval deficit in the patient’s mental lexicon: the intended concept is known, but the wrong, related label is produced. For the model, these substitutions introduce a subtle semantic drift that can be difficult to detect and correct, despite the surrounding context. In practice, this result tells us that semantic paraphasias of this sort consistently degrade the LLM’s output, underscoring the need for targeted strategies such as enhanced semantic error detection or fine-tuning on paraphasic patterns to improve corrective performance in aphasic speech.

The varying performance of LLMs across aphasia subtypes highlights both the nature of the underlying speech errors and the current model limitations. In our GLM analysis, we found that phonological substitutions (both real-word and non‐word), semantic paraphasias (related and unrelated substitutions), and neologisms (with known and unknown targets) each exerted a highly significant negative impact on reconstruction accuracy (all Wald χ^2^ > 9.8, *p* ≤ .007). These error types inherently disrupt the model’s contextual embedding process, neologisms introduce novel tokens outside the training distribution, phonological substitutions mimic spelling mistakes that LLMs cannot reliably correct, and semantic paraphasias obscure the intended meaning. While the model can often resolve minor semantic slips by leveraging conversation history, these more severe distortions consistently degrade its outputs. Together, these findings underscore the need for more specialised, fine‐tuned approaches such as targeted prompt engineering, subword or phoneme‐level embeddings, and error‐aware attention mechanisms to strengthen LLM robustness to the most challenging aphasic error patterns.

This multifaceted evaluation approach provides insights into the capabilities and limitations of LLMs in addressing both semantic and grammatical aspects of therapeutic conversations, contributing to a comprehensive assessment of their reliability in sentence reconstruction for aphasic speech. Moreover, the higher accuracy of the GPT-4o model suggests that newer and more complex models may be better equipped to handle impaired speech. With more fine-tuned and advanced models, there could be potential to position generative AI as a robust assistive tool for people with aphasia.

## Discussion

With the expanding landscape of generative AI, people are increasingly discovering tools to simplify their lives^36^. This is particularly evident for individuals with physical, sensory and cognitive impairments who utilise AI to enhance their daily activities. Over recent years, LLMs have garnered attention from researchers, practitioners, and the general public^21^. The inherent capabilities of LLMs in text generation, context understanding, and constructing meaningful and coherent language represent a significant advancement in AI solution development^37^. LLMs fundamentally possess the ability to predict missing words and fill language gaps in a contextually relevant manner, an especially pertinent function for people with language impairments. However, the translation of this technology to assist people with communication disability such as aphasia still needs to be explored and is in its infancy.

Recent research studies adopting this approach have indicated that future investigations should explore the potential of incorporating larger datasets and reliable measures of text generation tailored explicitly to the aphasia population^18,38^. Emphasis should also be placed on using naturalistic datasets over specific task-based conversation data, such as story retelling, to enhance the model’s generalizability in regular daily conversations. Considering these recommendations and inspired by the potential of generative AI to support individuals with aphasia, we proposed an innovative intelligent conversation framework built using OpenAI’s GPT-4o model and the Langchain framework. The Langchain framework facilitates a natural sequential flow of conversation and incorporates the retention of previous conversations through a conversation buffer memory. This capability enables the generation of more contextually relevant sentences based on prior interactions, as the LLM can understand the context of previous responses based on the self-attention mechanism in the transformer architecture.

**Fig. 6:**
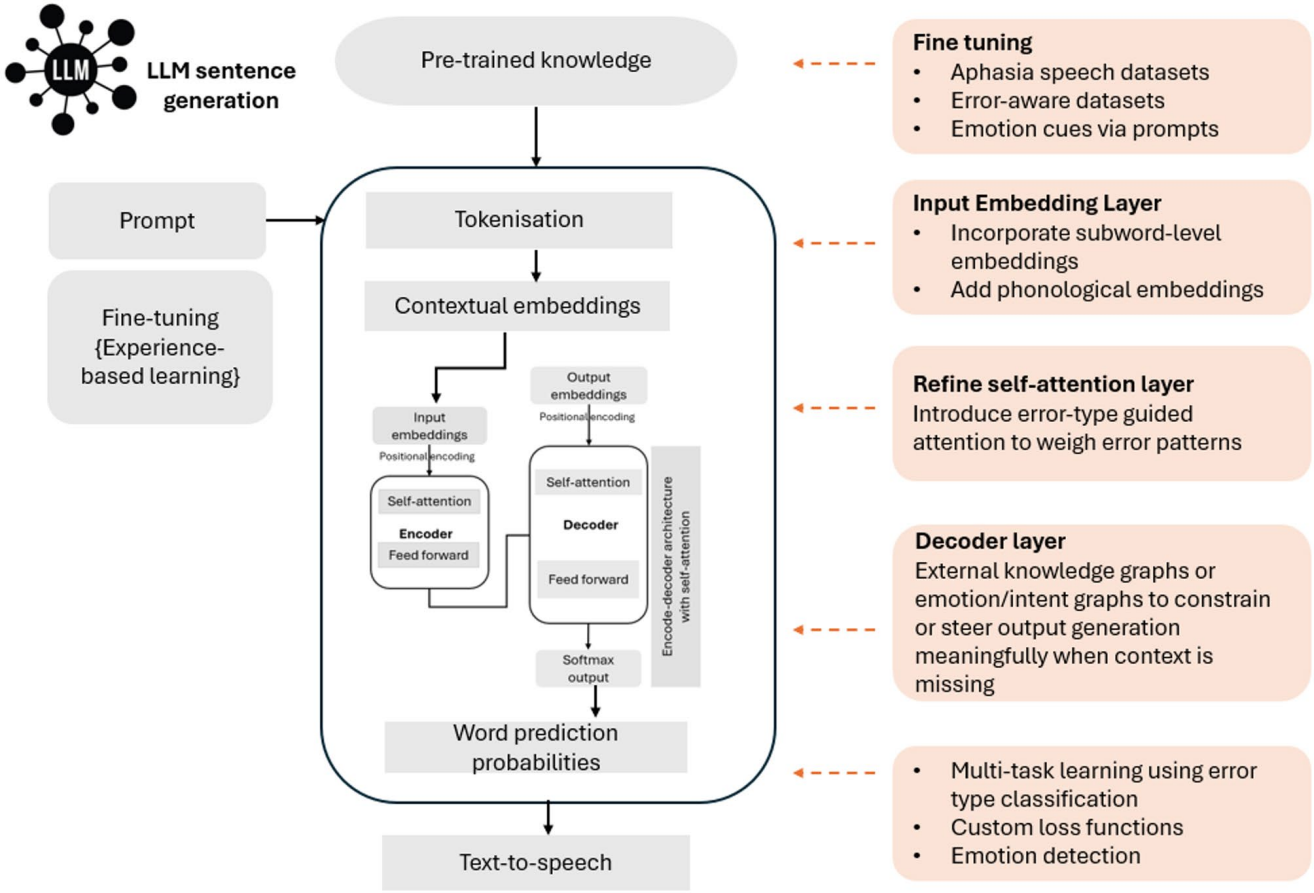
Proposed conceptual framework for LLMs to improve language error detection in Aphasia.

We further explored the capabilities of LLMs in impaired language correction based on different aphasia subtypes using the parallels between human speech production and LLM-based speech reconstruction (Fig. 1). This analysis highlights the strengths and limitations of LLMs in handling various language error types, especially in the context of speech or language impairments. The findings on different reconstruction accuracies and aphasia types align with the literature on aphasia type severity. It was shown that milder aphasia types benefit more from LLM-based assistive communication. Even with severe aphasia, the proposed approach maintained a high level of accuracy. As evidenced by improved accuracy in the GPT-4o model, AI research has the potential to be further expanded to accommodate nuances of speech errors in future to assist people with aphasia better. To enhance the performance of LLMS in processing aphasic speech, particularly challenging cases involving phonological errors, unknown target neologisms, semantic errors, and highly fragmented utterances, Fig. 6 illustrates potential design directions for future model adaptation based on our findings.

First, fine-tuning LLMs on aphasia-specific datasets, rich in labelled error types, is essential to enable generalisation and robust performance in this domain. The prompting stage could be improved via affective cues that are captured using other modalities. Second, the input embedding layer can be improved by incorporating subword or phoneme-level embeddings, allowing the model to better handle malformed (due to phonological errors) or non-standard words common in aphasia. Third, the attention mechanism could be adapted to tolerate fragmented syntax and guided by error types to more effectively focus on meaningful segments of speech. Positional encoding must also become more flexible to accommodate disordered sentence structures typical in agrammatic speech. Fourth, the decoder component can be enhanced by integrating external knowledge graphs or emotion-aware constraints to guide output generation when context is missing. Additionally, the loss function could be refined to penalise semantic deviation more than syntactic variation, and multi-task learning objectives could be introduced to train for correction and error classification jointly. An error classification module that can detect the type of errors could be introduced for this purpose. Detecting additional information such as emotions in conversation can further enhance the ability to learn the context, thereby resulting in higher accuracy. Together, these targeted improvements will better align LLMs with the cognitive-linguistic needs of people with aphasia and support more accurate, human-centric language reconstruction.

From a technological standpoint, our approach showcases the practical feasibility of integrating large language models to address communication challenges. The proposed system represents a significant potential advancement in digitized assistive communication. By leveraging memory updating mechanisms within LangChain, the system can maintain context across interactions, ensuring a more natural conversational flow and more accurate target selection. Unlike traditional assistive tools that rely on static phrase banks, LLMs dynamically identify potential topics the patient is discussing and adapt responses accordingly. This could be incorporated into mobile applications, chatbots or an assistive communication device to support people with aphasia in their daily communication. This sets a precedent for developing customised assistive technologies using generative AI that cater to the unique needs of individuals facing language-related issues beyond aphasia.

The practical implications of our proposed intelligent conversation assistant for language reconstruction in individuals with aphasia using large language models are manifold. From a clinical perspective, implementing this technology may become a tangible and efficient tool for speech pathologists and healthcare professionals working with individuals affected by aphasia. The system’s ability to correct neologisms in speech, fill word-finding gaps, and suggest sentence completions could significantly enhance communication outcomes. Such systems could improve societal participation and inclusion for individuals with aphasia in various aspects of life, enabling individuals with language impairments to participate more actively in social interactions, professional settings, and personal relationships.

While our intelligent conversation assistant exhibits promising results, certain limitations merit consideration. First, this and other recent studies rely on transcripts, whereas a practical end product will require rapid and accurate automatic speech detection. Dependency on the quality and diversity of training data also poses challenges and generalising the system to various aphasia types requires further exploration. Second, while our approach focuses on enhancing intelligibility and supporting clearer message reconstruction for individuals with aphasia, we acknowledge that the model may omit certain communicative markers, such as hesitations, repetitions, or pauses, which can carry meaningful pragmatic functions (e.g., indicating uncertainty or emphasis). These features, though atypical in conventional language models, are part of natural expression in many PWA utterances. However, our goal is to assist people whose speech is regularly interrupted due to impairments, and for whom conveying intended meaning clearly and promptly is paramount. We argue that the benefits of increased fluency and listener comprehension outweigh the occasional loss of such markers. Future research should prioritise personalisation and adaptability by tailoring the system to individual language patterns and exploring adaptive learning mechanisms. Incorporating user feedback will be essential to enhance model reliability and preserve each person’s unique communication style. Additionally, by continuously learning from user interactions, the system can develop an individualised profile of speech errors, enabling more precise and targeted support. This personalised approach enhances communication effectiveness, making AI-driven assistance more intuitive and user-centric.

Further refinements could include integrating demographic and personal information for deeper customisation based on an individual’s background, preferences, and communication needs. Expanding to additional modalities, such as speech, facial expressions, and gestures, could also significantly improve communication accuracy. Longitudinal studies will be necessary to assess the system’s long-term efficacy and user satisfaction, while clinical validation with healthcare professionals will ensure alignment with therapeutic goals.

Lastly, extending the application to address diverse language impairments could broaden the system’s impact and relevance within assistive technology.

## Supplementary Information

Below is the link to the electronic supplementary material.

Supplementary Material 1

## Data Availability

All transcripts used in the experiments are available via AphasiaBank (https://aphasia.talkbank.org/) Researchers, educators, and clinicians working with aphasia who are interested in joining the consortium should send an email to Abigail Medvic at amedvic@andrew.cmu.edu with contact information and affiliation to obtain access to this repository.
