# Supplementary material for: Reconstructing impaired language using generative AI for people with aphasia
Source: Sci Rep. 2025 Nov 19;15:40877. doi: 10.1038/s41598-025-24725-x (PMC12630810; doi:10.1038/s41598-025-24725-x)
Supplement: Supplementary file 1 — Supplementary Material 1 [file 41598_2025_24725_MOESM1_ESM.docx]

**Supplementary material**

Paired t-tests comparing BERTScore and cosine similarity on the same items to gauge if there is a difference between using these two metrics.

For each metric, the table reports the mean ± 95% CI, along with the paired mean difference with 95% CI, the t statistic and two-sided p-value.

| A : Cosine similarity |
| --- |
| B: BERTScore |

| Mean(A) | 0.86062 |
| --- | --- |
| Mean(B) | 0.85706 |
| Mean diff (A-B) | 0.00356 |
| Std diff | 0.02564 |
| t (paired) | 2.79210 |
| p (two-sided) | 0.00549 |
| 95% CI low | 0.00105 |
| 95% CI high | 0.00607 |
